# Complementary Muscle Metabolomics and Proteomics of Muscle in Cows With Post‐Calving Ketosis

**DOI:** 10.1111/jvim.70214

**Published:** 2025-09-30

**Authors:** Tao Tang, Jing Zhou, Jiahao Shao, Meigui Wang, Siqi Xia, Wenqiang Sun, Xianbo Jia, Jie Wang, Songjia Lai

**Affiliations:** ^1^ College of Animal Science and Technology Sichuan Agricultural University Chengdu Sichuan China; ^2^ Farm Animal Genetic Resources Exploration and Innovation Key Laboratory of Sichuan Province Sichuan Agricultural University Chengdu Sichuan China

**Keywords:** ketosis cow, metabolomics, proteomics, skeletal muscle

## Abstract

**Background:**

The muscle tissue of dairy cows is a site of β‐hydroxybutyrate (BHBA) metabolism. The mechanisms underlying the changes in proteins and metabolites in the muscle tissue of cows with ketosis remain unclear.

**Objectives:**

To elucidate the metabolic and physiological molecular adaptation mechanisms in the muscle tissue of cows with ketosis through metabolomics and proteomics.

**Animals:**

Six cows diagnosed with ketosis (CK, BHBA ≥ 2.4 mM) and six apparently healthy cows with BHBA < 1 mM (Con), all 1 week postpartum.

**Methods:**

Prospective cohort observational study. The content of BHBA in whole blood was detected by WST‐1 and TNN methods. HE staining was used to observe the physiological changes in the muscle tissue of cows with ketosis. Metabolomic analysis, proteomic analysis, and WB experiments were employed to identify differential metabolites and proteins.

**Results:**

The 685 metabolites and 356 proteins were identified to be differentially expressed in the CK group. In the muscle tissue of cows with ketosis, significant changes were observed in processes such as oxidative phosphorylation, PPAR signaling pathway, TCA cycle, amino acid degradation, and fatty acid oxidation. At the protein level, SDHA, SDHB, QCR6, QCR7, and IDH2 were significantly downregulated, while metabolites such as pyruvate were downregulated and BHBA was upregulated.

**Conclusions and Clinical Importance:**

The TCA cycle and mitochondrial respiratory chain in the muscle tissue of cows with ketosis might be impaired. These results provide valuable insights into the metabolic mechanisms of muscle tissue in cows with ketosis.

**Abbreviations**
BHBA: β‐hydroxybutyrate
BP: biological process
CC: cellular component
CK: clinical ketosis
Con: healthy controls
DMP: differently produced metabolites
DSP: differentially synthesized proteins
KEGG: Kyoto Encyclopedia of Genes and Genomes
MF: molecular function
NEB: negative energy balance
NEFA: non‐esterified fatty acids
ROS: reactive oxygen species

## 1 Introduction

After calving, due to the imbalance between energy intake and expenditure, dairy cows enter a state of negative energy balance (NEB) [1]. To adapt to NEB, the cows mobilize stored body fat, which is converted into triglycerides (TG). These TG are then broken down into non‐esterified fatty acids (NEFA) [2]. Additionally, when NEFA undergoes incomplete oxidation in the liver, a large amount of β‐hydroxybutyrate (BHBA) is produced and released into the bloodstream. This compound is then oxidized by specific organs, such as the heart, brain, and muscles, to provide energy [3]. However, when excessive BHBA is produced from NEFA due to inadequate metabolic adaptation, and this production exceeds the cow's ability to metabolize it, the concentration of BHBA in the blood can rise above 1.2 mmol/L, resulting in ketosis [4, 5]. The assessment of economic losses indicates that while ketosis is not the primary factor affecting milk production, it increases the risk of other diseases and culling, such as reduced milk yield and mastitis, and high prevalence is a key factor in reducing the economic efficiency of dairy cows [6, 7, 8].

Metabolomics technology is a powerful tool for elucidating the causes of diseases, determining disease diagnosis, and detecting and monitoring biomarkers [9, 10]. Proteomics represents the cumulative effects of gene functions and has become an important tool for exploring complex biological processes [11]. Additionally, proteomics provides a comprehensive view of protein expression patterns within an organism, which is crucial for identifying new biomarkers and pathways involved in various biological processes [12, 13, 14]. However, proteomic and metabolomic analysis methods have not yet been used to understand the regulatory mechanisms of muscle tissue in dairy cows with ketosis, as well as the changes in proteins and metabolites. Therefore, a better understanding of the metabolic products and protein characteristics in the muscles of cows with postpartum ketosis will help reduce the risk of ketosis and improve the profitability of dairy cows.

The combined analysis of these two methods is essential for enhancing our understanding of cellular signaling networks, revealing the intricate dynamics of protein‐metabolite interactions across different cellular states, and improving our diagnostic capabilities as well as molecular insights into potential disease mechanisms. In this study, we hypothesize that the metabolomic and proteomic profiles of muscle tissue from cows diagnosed with clinical ketosis will differ from those of healthy controls. By employing liquid chromatography‐mass spectrometry (LC/MS) based metabolomics and proteomics approaches, we aim to gain a comprehensive understanding of the metabolic patterns in the muscle tissues of cows with ketosis, while also providing preliminary insights into the metabolic mechanisms related to BHBA. Through the integration of metabolomics and proteomics data, we can identify key regulatory factors associated with BHBA metabolism in the muscle tissues of ketosis cows and construct critical protein‐metabolite networks.

## 2 Materials and Methods

### 2.1 Experimental Animals and Muscle Tissue Samples

The Institutional Animal Care and Use Committee of the College of Animal Science and Technology, Sichuan Agricultural University, China, approved all experimental procedures using cows in our study (Certification No. SYXK2019‐187) The experiment was conducted at a modern dairy farm in Mianyang, Sichuan Province. We selected 70 cows that were all in their third lactation and had due dates within ±7 days, ensuring they had similar body condition scores (Table S1, BCS = 2.75–3.5, Chinese Holstein dairy cows).

The experimental cows were raised in the same environment, with identical food, straw used as bedding, free access to fresh water, and consistent management practices. During the dry period, TMR was fed twice a day at 11:00 and 19:00. After calving, TMR was fed three times daily at 06:00, 14:00, and 23:00. The composition and ingredients of the feed are detailed in the study by Wu [15] et al. For each cow, ketosis was diagnosed at postpartum (between 1 and 6 days) according to blood concentration of BHBA. The blood samples from the tail vein of dairy cows with 1–2 h before feeding were collected, and the BHBA levels of the dairy cows were measured three times using the TNN (Beijing Yicheng, China) handheld BHBA meter. Cows with clinical ketosis have a blood BHBA ≥ 2.40 mM, while healthy cows have a BHBA < 1.00 mM. Ultimately, 12 cows were selected from the herd, with 6 being clinically ketosis designated as the CK and 6 serving as healthy controls designated as the Con. Neither the CK nor the Con had any other diseases. The Gluteus maximus muscle samples were collected from 6 CK cows and 6 Con cows using a disposable semi‐automatic biopsy device (Promisemed, Zhejiang, SNB‐2015C). Six samples were taken from each cow, with three samples from the left Gluteus maximus and three from the right. After collection, the tissue samples were immediately placed in cryovials and stored in liquid nitrogen for subsequent omics analysis.

Significantly, the cows diagnosed with ketosis received prompt treatment immediately after the collection of blood and muscle tissue samples. The clinical symptoms observed in the cows included in this study were generally mild, with only a few individuals presenting with abomasal displacement. The primary treatment protocol consisted of oral administration of propylene glycol and glucose solution twice daily, in the morning and evening. For cows with abomasal displacement, intravenous glucose infusion was administered in addition to oral glucose supplementation, along with appropriate vitamin and mineral support as necessary. During the subsequent days, blood BHBA concentrations were continuously monitored, and clinical observations were performed to evaluate the progression of recovery.

### 2.2 Detection of BHBA Content Using the WST‐1 Method and HE Staining

The laboratory conducted further testing on the whole blood samples selected by TNN using the β‐hydroxybutyrate (β‐HB) content measurement kit (Solarbio, Beijing, bc5085). The detection of BHBA content is strictly performed according to the instructions of the kit. In simple terms, it involves the following steps: Collect the blood samples from the dairy cows to be tested, and sequentially add specific reagents according to the instructions. Incubate the mixture at the specified temperature and time to complete the reaction. Measure the absorbance of the reaction solution at 450 nm using a microplate reader. Finally, calculate the BHBA content in the serum based on the formula [16].

Muscle tissue samples underwent dehydration, clearing, paraffin embedding, and sectioning, followed by the hematoxylin and eosin stain (H&E) method. Histopathological changes were examined under a microscope (OLYMPUS, Japan, BX53F), allowing for a comprehensive analysis of the entire tissue section. The cross‐sectional area of muscle fibers in the CK and Con groups (six images per group, 200×) muscle tissue images obtained through the microscopic imaging system was analyzed using Image J (v 1.53, Bethesda, MD, USA).

### 2.3 Liquid Chromatography‐Mass Spectrometry Metabolomics Analysis

The extraction of metabolites, The LC–MS analysis was performed using the Ultim3000 high‐performance liquid chromatography system coupled with the Orbitrap Exploris 480 high‐resolution mass spectrometer (Thermo, USA), and data preprocessing and annotation were similar to previous studies [17]. Briefly, weigh 50 mg of samples from the CK and Con, add 1000 μL of extraction solution containing internal standards, and vortex for 30 s to extract metabolites. Equal aliquots of extract liquid from all experimental samples were pooled as quality control (QC) specimens. The column used is purchased from Waters Acquity UPLC HSS T3 column (1.8 μm 2.1 × 100 mm; Waters Corporation, USA) [18]. The original data file acquired by LC–MS was imported by CD search Library software (https://www.ncbi.nlm.nih.gov/Structure/cdd/wrpsb.cgi). Peak extraction, peak alignment, m/z and retention time correction were respectively performed by the program. The conditions for peak extraction were set as follows: mass deviation of 5 ppm, signal intensity deviation of 30%, S/*N* ≥ 3, signal intensity ≥ 100,000and ion addition. Finally, metabolic identification information was obtained by searching the mzCloud (https://www.mzcloud.org), mzVault (https://www.mzvault.org), and ChemSpider (https://www.chemspider.com) databases in our laboratory, and theoretical fragments were integrated and quantified.

### 2.4 Liquid Chromatography‐Mass Spectrometry Proteomics Analysis

The process begins with the extraction and digestion of the sample proteins. To the sample, 300 μL of 8 M urea and a protease inhibitor were added. The mixture was centrifuged at 14,000*g* for 20 min, and the supernatant was collected. Protein concentration was determined using the Bradford method. The proteins were then reduced by adding 200 mM dithiothreitol (DTT) solution and incubating at 37°C for 1 h. Next, 50 mM ammonium bicarbonate (ABC) buffer was added to dilute the sample eight times. Finally, trypsin was added, and the sample was incubated overnight at 37°C. Secondly, the protein solution was separated using Reversed‐Phase Liquid Chromatography (RP‐HPLC); peptide identification of the separated protein solution was performed using LC–MS/MS by RIGOL L‐3000 High performance liquid chromatography system (RIGOL Technology, Beijing, China).

### 2.5 Western Blot Analysis

The protein samples used in western blotting are identical to those employed in proteomics analysis. Boil the protein samples at 99°C for 10 min. Then, separate 35 μg of total protein per sample by SDS‐PAGE and transfer them to a PVDF membrane (0.22 μm, Merck Millipore) using the wet transfer method (400 mA, 60 min). Incubate gently at room temperature for 2 h in Tris‐buffered saline with Tween (TBST; 150 mM NaCl, 50 mM Tris, pH 8.8, 0.1% Tween 20) containing 5.0% NON‐Fat powdered milk (Sangon, Shanghai, JC28BA0010). After washing the membrane three times with TBST, incubate it at 4°C for 12 h in PBS containing the primary antibody. The specific details of the primary antibody are provided in Table S2. Subsequently incubated with the secondary antibodies for 2 h (Goat anti‐rabbit IgG H&L [HRP], Zen bioscience, Chengdu, China [antibody: PBS = 1:10000]). Subsequently, the membranes were washed three times. The membrane's immunoreactivity was detected using ECL chemiluminescence reagent (HAKATA, Shanghai, China). Images were captured using the Touch Imager Pro from e‐BLOT Life Sciences (Shanghai, China). The MB and PPARγ proteins were normalized using β‐actin as the housekeeping gene, while FABP4, SOD3, SQSTM1, IDH2, and MYH7 were normalized using GAPDH as the housekeeping gene.

### 2.6 Data Processing, Bioinformatics, and Statistical Analysis

The metabolomics analysis process was similar to previous studies [19]. Principal component analysis and Spearman correlation analysis were used to assess the reproducibility of samples within the group and quality control samples. According to the grouping information, the difference multiples, T test was used to calculate the difference significance *p* value of each compound. The variable importance in projection (VIP) value of the model was calculated using multiple cross‐validation. The method of combining the difference multiple, the *Padj*, and the VIP value of the OPLS‐DA model was adopted to screen for significantly differently produced metabolites (DPM). The screening criteria are FC > 1, *p* < 0.05, and VIP > 1. The different metabolites of KEGG pathway enrichment significance were calculated using the hypergeometric distribution test. In proteomics analysis, the student's *t* test is used to compare protein differences between groups and calculate *p* values. To compare proteins between different treatments, a *t* test was used to determine the *p* value. A fold change (FC) of 1 and a false discovery rate (FDR) corrected *p* < 0.05 were set as the threshold for identifying differentially synthesized proteins (DSP). Overall, DSP were examined to enrich Gene Ontology (GO) terms, cellular component (CC), molecular function (MF), and biological process (BP). Protein–protein interaction network was visualized and analyzed utilizing STRING V.11.0. A network relationship between differential proteins was constructed using Cytoscape. For the Western blot data, a t‐test was used to compare the differences in parameters between the treatment groups, and the *p* value was calculated. When *p < 0.05, the differences were significant.

## 3 Results

### 3.1 Determination of BHBA in Periparturient Dairy Cows and Collection of Muscle Tissue for HE Staining

**FIGURE 1:**
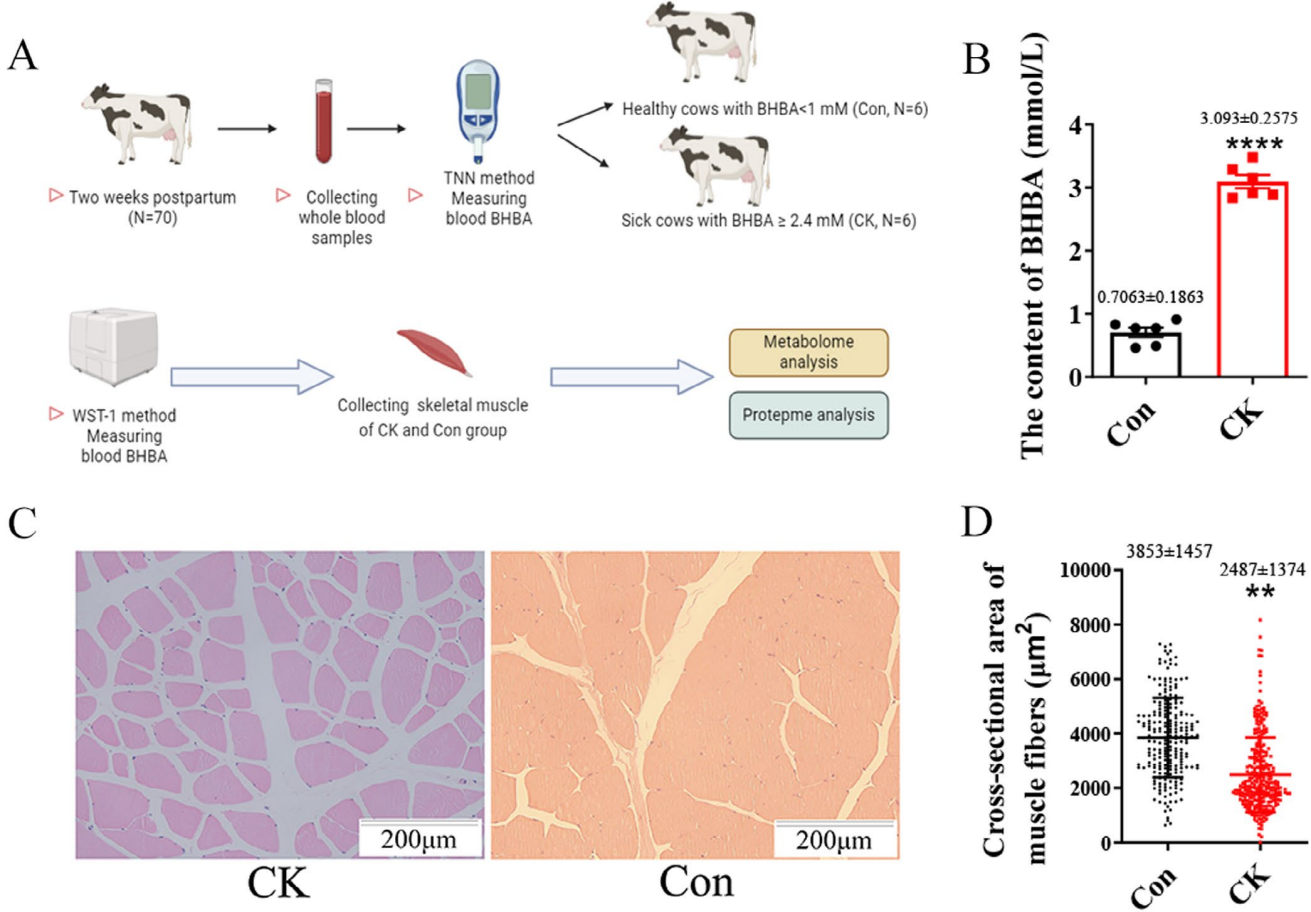
Experimental design and determination of BHAB concentration and HE staining of muscle tissue (200×). (A) Experimental design and sample collection. (B) The concentration of BHBA was determined by WST‐1 (the numbers in the bar chart represent the mean ± SD). (C) Muscle tissue was stained with HE (200×). (D) Muscle fiber cross‐sectional area statistics (*n* = 6; the numbers in the bar chart represent the mean ± SD).

The concentration of BHBA in the serum of postpartum cows was measured using a handheld TNN device, and the cows were grouped according to BHBA concentrations. Cows with a serum BHBA concentration ≥ 2.40 mM were classified into the CK (*n* = 6), while those with a BHBA concentration < 1.00 mM were classified into the Con (*n* = 6; Figure 1A). The BHBA levels in the serum of the CK and Con were re‐measured using the WST‐1 method. The results showed that the BHBA concentration in the Con was less than 1 mM, while the CK had a concentration greater than 2.4 mM, which meets the experimental requirements for subsequent proteomic and metabolomic analyses (*p* < 0.01; Figure 1B). The H&E staining results of the muscle tissue showed (Figure 1C) that the muscle fibers in the Con group were arranged neatly, exhibiting a typical striated structure. The morphology of the muscle fibers was normal, with a uniform number of nuclei and no lesions observed. The muscle fibers in the CK group are irregular in size, with larger gaps, and multinucleated muscle fibers are present, suggesting that the muscle tissue in ketotic cows might have undergone damage and pathological changes. Additionally, statistical analysis of the cross‐sectional area of the muscle fibers revealed a significant reduction in the CK (*p <* 0.01; Figure 1D).

### 3.2 Metabolite Profiles Protein of Muscle Samples and Data Analysis

**FIGURE 2:**
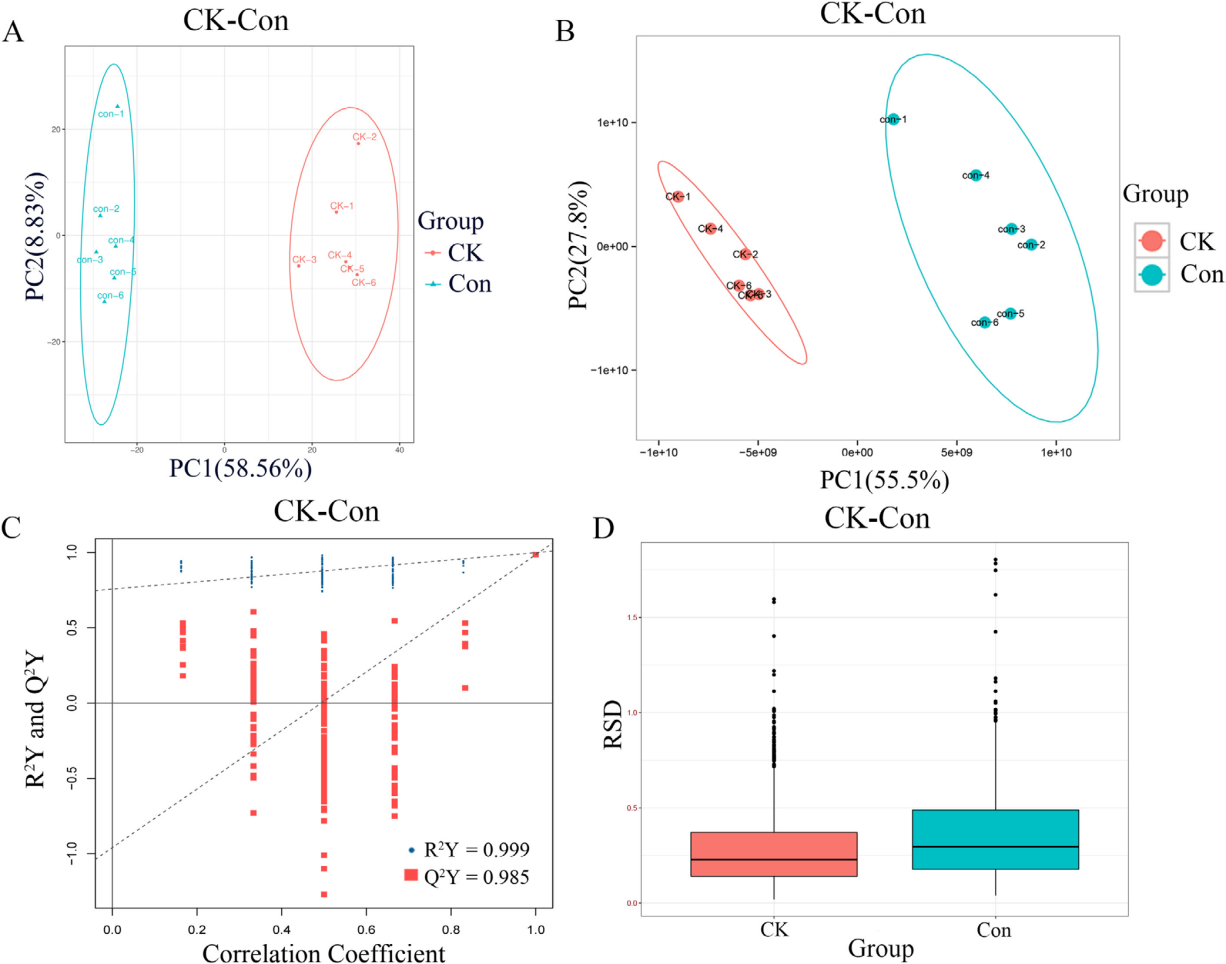
Quality control of proteomic and metabolomics data. (A) Principal component analysis (PCA) of metabolites in the Con and CK groups in the metabolomics. (B) PCA of proteins in the Con and CK groups in the proteomics. (C) OPLS‐DA analysis in the metabolomics. (D) Distribution of relative standard deviation (RSD) for Con and CK groups in proteomics.

Based on the Orbitrap LC–MS platform, a qualitative and quantitative analysis of metabolites was conducted on a total of 12 samples from the Con and CK groups. This report presents the metabolites detected under default conditions, with a total of 1342 peaks identified, of which 1252 metabolites were annotated (Table S3). The signal intensity integration method using peptide segments was employed to quantify a total of 12 experimental samples of Con and CK, resulting in the detection of 933 protein information (Table S4). The two groups of samples are well separated, and samples within the same group are well clustered in the PCA score plot (Figure 2A,B). The QC specimens overlap well in the PCA score plot, indicating that the metabolomics methods used are robust, repeatable, and stable. In the metabolomics analysis of Con versus CK, the equivalent *R* ^2^ *Y* value of the OPLS‐DA model is 0.999, and the intercept from the permutation test is 0.985, indicating that the model has good validity for identifying differences between the two treatments (Figure 2C). In the proteome, the range of RSD is within 2%, indicating that the proteomic data is stable and reliable, and the reproducibility of biological replicates within the group is better (Figure 2D).

### 3.3 Differential Metabolite and Pathway Analysis

**FIGURE 3:**
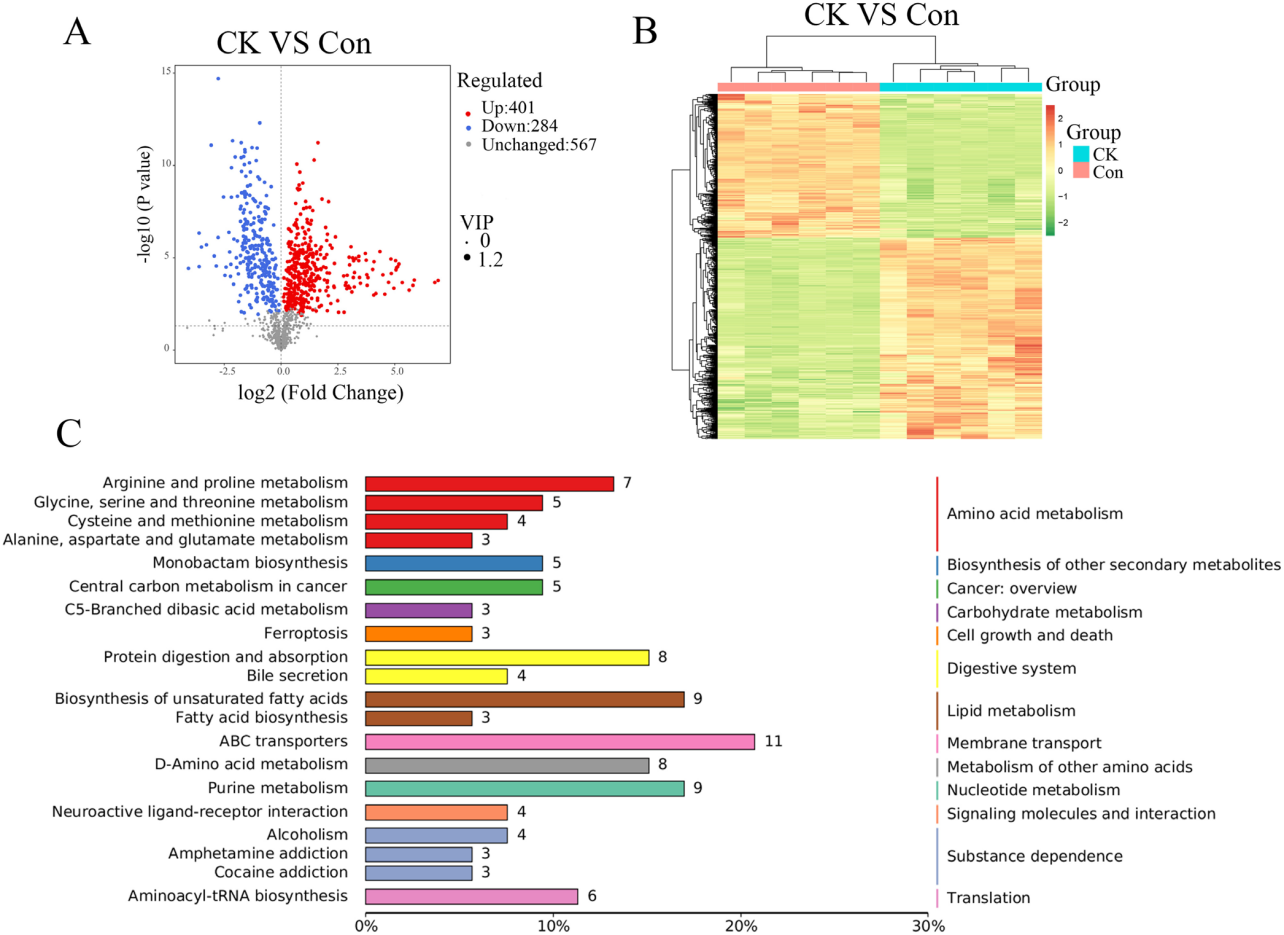
Analysis of differential metabolites in metabolomics: Con versus CK. (A) Volcano plot of differential metabolites between Con and CK under default mode (*n* = 6). The horizontal axis represents the differences in multiple changes of metabolites between different groups (log2 fold change), while the vertical axis indicates the significance level of the differences (−log *p* value). Each point on the volcano plot represents a metabolite. Significantly upregulated metabolites are represented by red dots, and significantly downregulated are represented by blue dots and without significance are represented by gray dots. The size of the points corresponds to the VIP value. (B) Differential Metabolite Clustering Heatmap in Con and CK groups. (C) Perform KEGG pathway analysis on the differential metabolites identified in muscle biopsy tissues of Con and CK, *x* axis percentage is fraction of the enriched items relative to total number of items in the pathway. The entries under the same box in the figure represent the hierarchical classification annotations of KEGG pathways, corresponding to KO pathway level 2 and KO pathway level 3. The length of the bars indicates the number of metabolites annotated in that pathway.

Under the criteria of FC > 1, VIP ≥ 1.0, and *p* < 0.05, a total of 685 DPM were identified, with 401 increased and 284 decreased in the CK group (Table S5, Figure 3A). The generated clustering heatmap shows that the distribution of metabolites in the Con and CK groups is similar, while the dendrogram indicates that the samples from the experimental group and the control group can be separated (Figure 3B). Use the KEGG database to annotate the differential metabolites and select the top 20 entries with the highest number of annotated differential metabolites in the pathways (Figure 3C). The AA, lipid, energy, carbohydrate and nucleotide metabolism were mainly involved in these pathways.

### 3.4 Differential Protein and Pathway Analysis

**FIGURE 4:**
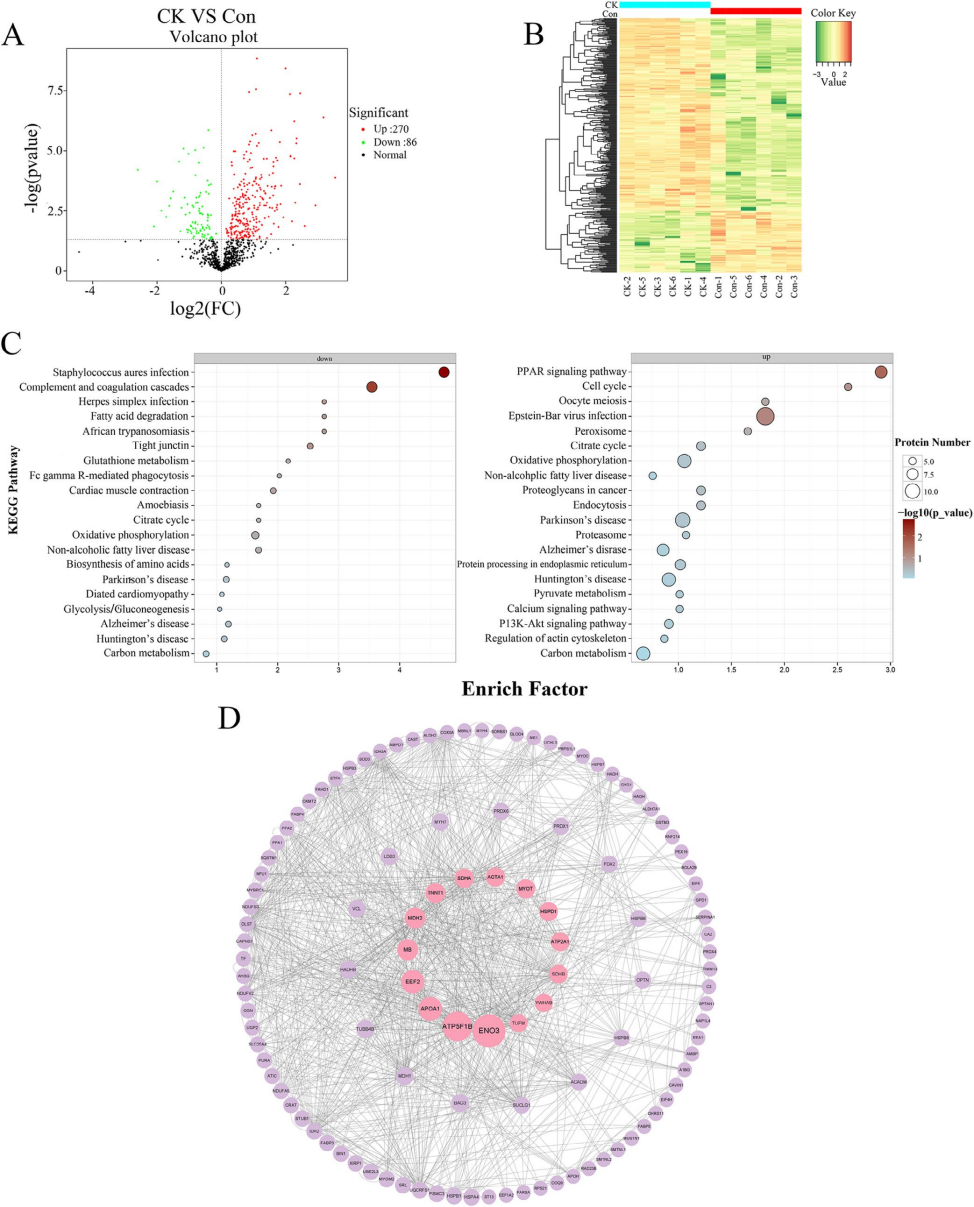
Differential protein and pathway analysis. (A) Volcano plot of differential protein between Con and CK (*n* = 6). (B) Differential Metabolite Clustering Heatmap in Con and CK groups. (C) KEGG enrichment analysis of differentially up‐regulated (Right) and down‐regulated (Left) proteins. The size of the circles represents the number of differential proteins, while the color of the circles represents −log(*p* value). (D) Protein–protein interaction (PPI) network analysis of the DSP. The circle size represents the betweenness centrality (BC).

**FIGURE 5:**
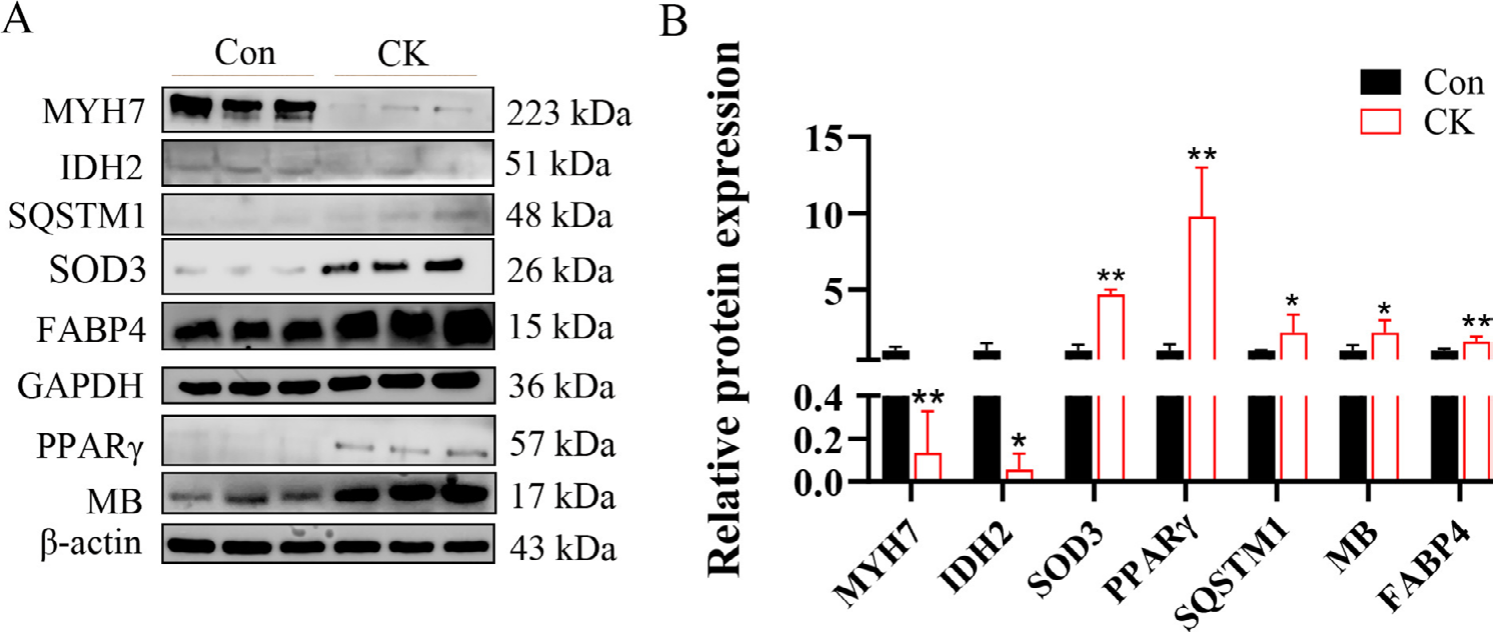
The expression pattern of differential proteins in the muscle tissue of periparturient ketosis dairy cows (*n* = 3). (A) Western blot results of some differential proteins in the muscle tissue of ketosis dairy cows. (B) Relative selected protein; β‐actin(PPARγ, MB) and GAPDH (FABP4, SOD3, SQSTM1, IDH2, MYH7) protein levels were calculated by a grayscale scan. Data are expressed as mean with 95% confidence interval.

Under the criteria of FC > 1 and *p* < 0.05, a total of 685 DSP were identified from CK and Con groups, with 270 increased and 86 decreased in the CK group (Table S6, Figure 4A). Based on the protein expression data of 356 DSP, the two groups are well‐separated in clustering (Figure 4B). These up and down proteins were enriched into 165 pathways through KEGG pathway analysis, of which five paths were significant (−log(*p* value) > 1.3, Figure 4C), an example is the PPAR signaling pathway. Notably, the proteins were enriched in KEGG analysis, namely TCA cycle, fatty acid degradation, oxidative phosphorylation, glutathione metabolism, peroxisome and so on. These results suggest that the fatty acid metabolism, mobilization, and energy metabolism, as well as oxidative phosphorylation, in ketosis cows are affected to varying degrees. In addition, 47 important DSP were mapped into these KEGG pathways (Table 1). The PPI network results indicate that these proteins are mainly involved in metabolic pathways such as the citrate cycle (TCA cycle), carbon metabolism, pyruvate metabolism, and oxidative phosphorylation (Figure 4D). The top 10 proteins with the highest betweenness centrality (BC) values include ATP5FB1, APOA1, EEF2, MDH2, SDHA, and MYOT. These proteins are involved in lipid metabolism and oxidative phosphorylation, suggesting that ketosis cows might be related to the fatty acid β‐oxidation involved in the mitochondrial respiratory chain, which could also affect the oxidative stress levels in the muscle tissue of cows. The Western blot results are consistent with the protein sequencing results for the selected seven differential proteins (Figure 5).

**TABLE 1:** Important differential synthesis proteins in muscle tissue of ketosis dairy cows (*n* = 6)^a^.

Gene name	Protein	Accession	log2FC	Trend^b^	*p* ^c^
APOH	Apolipoprotein H	A0A140T843	−0.806	−	< 0.01
SDHB	Succinate Dehydrogenase Complex Subunit B	A0A3Q1LZ07	−0.410	−	< 0.01
HAGH	Hydroxyacyl‐CoA dehydrogenase	A0A3Q1ND53	−0.423	−	< 0.01
ALDH7A1	Aldehyde Dehydrogenase 7 Family Member A1	A0A3S5ZP98	−0.427	−	< 0.01
ATP5F1B	ATP Synthase F1 Subunit Beta	A0A452DII8	0.692	+	0.001
GPD1	Glycerol‐3‐Phosphate Dehydrogenase 1	A0A452DJJ3	−0.550	−	< 0.01
SLC25A4	Solute Carrier Family 25 Member 4	A0A4W2BY60	1.161	+	< 0.01
SUCLG1	Succinate‐CoA Ligase Glycosylated Subunit 1	A0A4W2CBS2	1.048	+	< 0.01
UGP2	UDP‐Glucose Pyrophosphorylase 2	A0A4W2CCV6	0.735	+	0.001
PRDX6	Peroxiredoxin 6	A0A4W2CJ87	0.318	+	< 0.01
FABP5	Fatty Acid Binding Protein 5	A0A4W2CPJ9	0.570	+	0.002
NDUFA5	NADH: ubiquinone oxidoreductase subunit A5	A0A4W2CSL0	0.891	+	0.009
CRAT	Carnitine O‐acetyltransferase	A0A4W2CU46	0.777	+	0.024
TNNT1	Troponin T, Skeletal Muscle	A0A4W2D1S0	0.466	+	0.022
TIMM13	Translocase of Inner Mitochondrial Membrane 13	A0A4W2D6S0	2.264	+	< 0.01
SQSTM1	Sequestosome 1	A0A4W2DDV1	2.149	+	< 0.01
ACADM	Acyl‐CoA Dehydrogenase, Medium‐Chain	A0A4W2DHN1	0.400	+	0.025
SOD3	Superoxide Dismutase 3	A0A4W2DIK2	1.074	+	< 0.01
BIN1	Bridging Integrator 1	A0A4W2DPC0	0.333	+	< 0.01
ST13	Suppressor of Tumorigenicity 13	A0A4W2DUJ5	0.241	+	< 0.01
ETFA	Electron Transfer Flavoprotein Alpha Subunit	A0A4W2DX30	0.272	+	0.016
COQ9	Coenzyme Q9	A0A4W2E053	1.216	+	< 0.01
CKMT2	Creatine Kinase, Mitochondrial 2	A0A4W2E2M7	0.407	+	0.029
MAP2K6	Mitogen‐Activated Protein Kinase Kinase 6	A0A4W2E5A9	0.896	+	0.019
ME1	Malic Enzyme 1	A0A4W2E9T3	0.701	+	0.022
IDH3A	Isocitrate Dehydrogenase 3 Alpha	A0A4W2EG40	1.616	+	< 0.01
NDUFS3	NADH:ubiquinone oxidoreductase Fe‐S protein 3	A0A4W2EKS6	−0.404	−	< 0.01
HADH	Hydroxyacyl‐CoA Dehydrogenase	A0A4W2F1T0	−1.065	−	0.019
GLOD4	Glutamate Oxidase Domain Containing 4	A0A4W2FXR4	0.559	+	< 0.01
MYH4	Myosin Heavy Chain 4	A0A4W2G576	−1.578	−	< 0.01
DLST	Dihydrolipoamide S‐succinyltransferase	A0A4W2GWM3	0.702	+	< 0.01
FDX2	Ferredoxin 2	A0A4W2H1Z0	0.517	+	< 0.01
FABP3	Fatty Acid Binding Protein 3	A0A4W2HP58	0.972	+	< 0.01
DHRS11	Dehydrogenase/Reductase 11	A0A4W2I5W2	0.551	+	0.003
TUFM	Mitochondrial Elongation Factor Tu	A0A4W2I843	0.642	+	0.025
ATP2A1	Calcium ATPase 2A	A0A4W2IA03	0.807	+	0.023
OPTN	Optineurin	A0A4W2IAY1	3.535	+	< 0.01
FAHD1	Fumarylacetoacetate Hydrolase Domain Containing 1	A0A4W2INI1	0.580	+	0.025
FABP4	Fatty Acid Binding Protein 4	F1MHQ4	2.295	+	0.001
MYH7	Myosin Heavy Chain 7	F1MM07	−1.025	−	< 0.01
SDHA	Succinate Dehydrogenase Complex Subunit A	G5E6M7	−1.141	−	< 0.01
COX5A	Cytochrome c Oxidase Subunit 5A	P00426	0.805	+	0.008
NDUFV2	NADH Dehydrogenase (Ubiquinone) Fe‐S Protein 2)	P04394	0.524	+	0.009
IDH2	Isocitrate Dehydrogenase 2	Q04467	−0.338	−	0.047
MDH1	Malate Dehydrogenase 1	Q3T145	0.317	+	0.029
MDH2	Malate Dehydrogenase 2	Q32LG3	0.337	+	0.005
PRDX1	Peroxiredoxin 1	Q5E947	0.359	+	0.016

Abbreviation: log2FC, log2(fold change).

^a^ The table only shows the differentially synthesized proteins in significant pathways from the Kyoto Encyclopedia of Genes and Genomes (KEGG) database.

^b^ + and −: abundance increased and decreased in the CK group, respectively.

^c^ *p* values were determined utilizing the student's *t*‐test between the CK and Con groups.

### 3.5 Integrating Metabolomics and Proteomics Analyses

**FIGURE 6:**
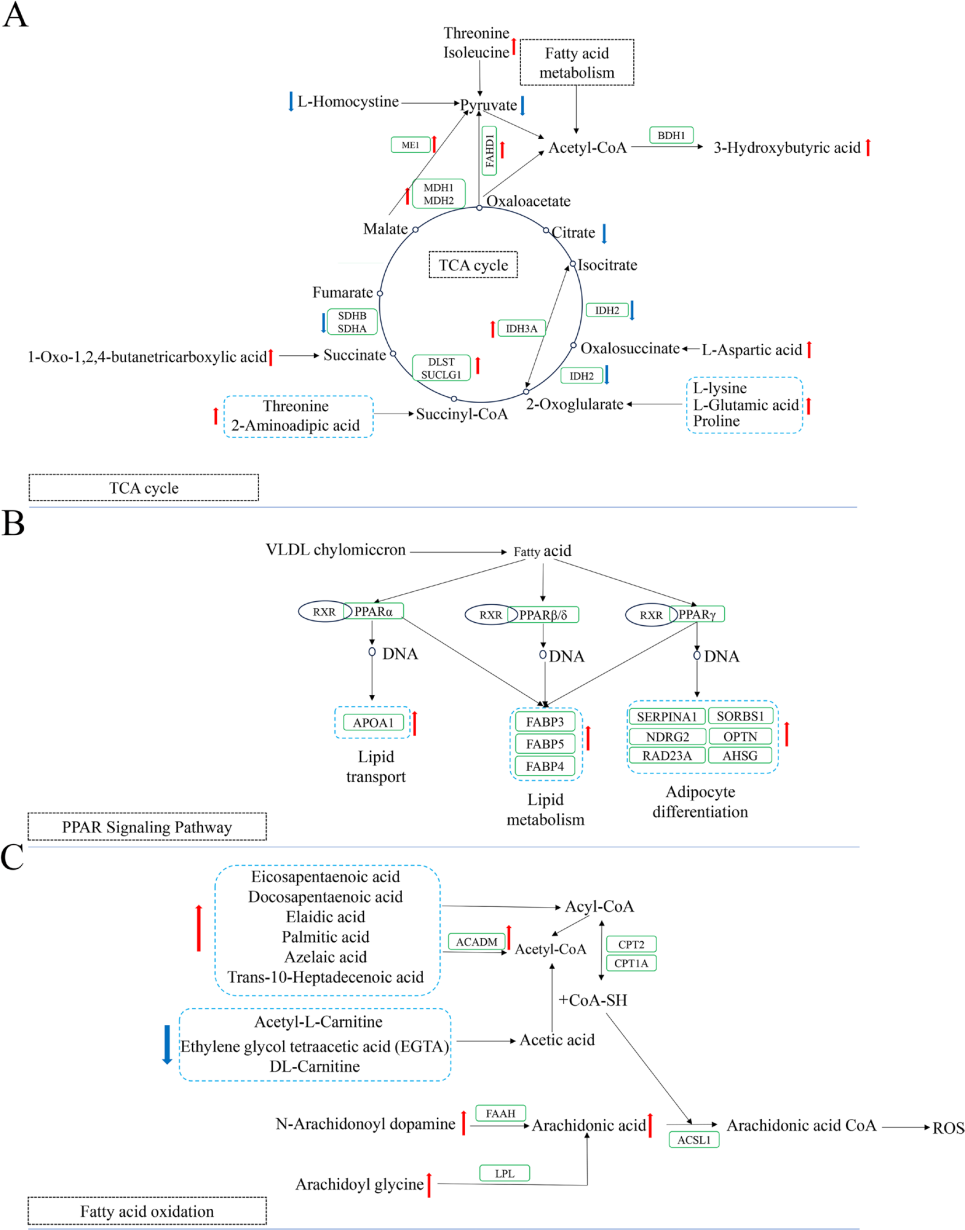
Integrating metabolomics and proteomics analyses. (A) Schematic diagram of the effects of different proteins and metabolites in muscle biopsies of cows with ketosis after calving on amino acid (AA), carbohydrate metabolism and energy metabolism. Schematic diagrams of the peroxisome proliferator‐activated receptor (PPAR) signaling pathway (B) and fatty acid oxidation (C). Please note that this is a hypothetical relationship based on current data. The green rectangles surround the proteins. Red arrows indicate upregulation in the CK group, while blue arrows indicate downregulation. The black dashed boxes represent metabolic processes, and black arrows indicate reaction processes. For specific protein names, refer to Table 1.

**FIGURE 7:**
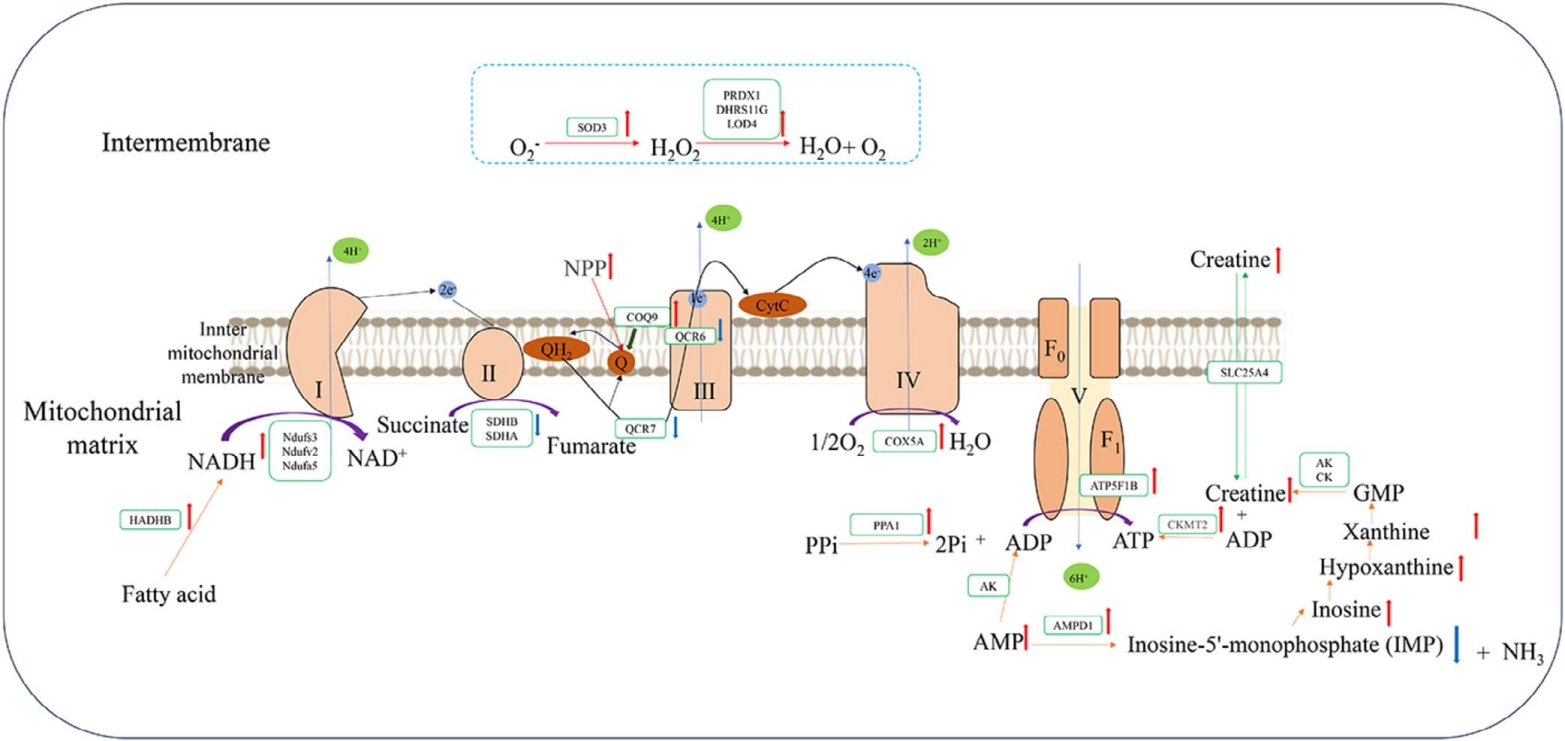
Effects of different proteins and metabolites in muscle biopsies of cows with ketosis after calving on oxidative phosphorylation. Red arrows indicate upregulation in the CK group, while blue arrows indicate downregulation. The black dashed boxes represent metabolic processes. Orange arrows indicate reaction processes. Black arrows show the direction of electron transfer, and blue arrows indicate the direction of H^+^ transfer. For specific protein names, refer to Table 1.

Using the KEGG Mapper tool, the DSP and DPM were mapped to the significantly altered KEGG pathways [20]. The identified pathways include carbohydrate metabolism, energy metabolism, lipid metabolism, amino acid metabolism, cell growth and death, arginine and proline metabolism, glycine, serine, and threonine metabolism, AMP‐activated protein kinase (AMPK) signaling pathway, pyruvate metabolism, and the tricarboxylic acid cycle (Figure 6). The key pathways associated with DSP and DPM mapping primarily cluster around amino acid metabolism, lipid metabolism, carbohydrate metabolism, oxidative status, oxidative phosphorylation respiratory chain status (Figure 7), and mitochondrial structure and function. The 35 DPM and 39 DSP are mainly involved in these pathways and have been identified as key components. These critical DPM and DSP with mapped pathways are manually linked together.

## 4 Discussion

This study conducted metabolomics and proteomics sequencing on the muscle tissue of ketosis cows within 1 week after calving, along with HE staining. Omics found that proteins such as SDHA, SDHB, QCR6, QCR7, and IDH2 were significantly downregulated, while metabolites like pyruvate were downregulated and BHBA, arachidonic acid, and others were upregulated. Additionally, combined with the HE staining results, it suggested that muscle tissue in ketosis cows might be damaged, the TCA cycle and mitochondrial respiratory chain might be impaired, and the oxidative stress state might be elevated.

### 4.1 Skeletal Muscle Injury of CK Cows

During the transition period, the imbalance between energy demand and energy intake in cows can lead to NEB. Additionally, the demand for glucose and metabolizable energy postpartum is higher than that before calving [21]. Moreover, in the 3–5 weeks before lactation, cows not only consume body fat but also utilize muscle protein to generate energy and provide the necessary amino acids for glycogenesis [22, 23]. However, the decrease in blood glucose levels in ketosis cows leads to excessive mobilization of the body's stored fat to maintain blood glucose concentration, which results in the production of large amounts of BHBA. BHBA is transported throughout the body via the bloodstream to the muscles, where it is broken down into water and CO_2_ to produce energy. However, this study found that the elevated levels of BHBA in muscle tissue might reflect the adaptation or stress response of muscle cells in energy metabolism, and it might also suggest that muscle tissue could be experiencing some damage or metabolic abnormalities due to this metabolic disorder. However, the specific mechanisms by which BHBA affects muscle tissue still need to be verified through subsequent experiments. Histological results from this study indicated that the gaps between muscle fibers widened, which might suggest that the muscle is undergoing damage or regeneration [24]. Additionally, an increase in the number of nuclei was observed, with some muscle fibers being bi‐ or tri‐nucleated, suggesting the possibility of inflammation in the muscle tissue of ketosis cows [25].

In skeletal muscle, different muscle fiber types perform distinct biological functions. Type I muscle fibers have higher activities of GPX, SOD, CAT, and other enzymes, which enable them to resist reactive oxygen species (ROS) produced by the body [26]. However, Type II muscle fibers are associated with contraction speed, ATPase activity, and Myonuclear Domain (MND) size, which are closely related to the size of the muscle fibers [27]. In this study, a decrease in the expression levels of MYH7 and MYH4 proteins was observed. MYH7 encodes β‐myosin, which is primarily expressed in Type I muscle fibers (slow‐twitch fibers), while MYH4 encodes IIx myosin, mainly expressed in Type II muscle fibers (fast‐twitch fibers). This suggests that the muscle fibers of cows with ketosis after calving are damaged. However, whether the damage to the muscle tissue in ketosis cows is caused by excessively high BHBA concentrations still needs to be verified through further experiments.

### 4.2 Carbohydrate Metabolism in the Muscle Tissue of CK Cows

Isocitrate dehydrogenase (IDH) catalyzes the oxidative decarboxylation of isocitrate, producing α‐ketoglutarate and CO_2_ [28]. IDH3 positively regulates the TCA cycle to increase energy metabolism [29]. IDH2, the NADP^+^‐dependent enzyme, generates NADPH during the conversion of isocitrate to alpha‐ketoglutarate, which is crucial for defending against ROS produced by mitochondrial respiration [30, 31]. In this study, the proteomic analysis revealed a downregulation of IDH2 protein expression and an upregulation of IDH3 expression. This might indicate that the muscle tissue of ketosis‐affected cows is undergoing oxidative stress, and the self‐adaptive mechanisms to combat oxidative stress are impaired. The upregulation of IDH3A also suggests that post‐calving cows have an increased energy demand, requiring a large amount of ATP. In addition, when the enzymatic activity of SDHA is reduced, it can hinder the TCA cycle, leading to insufficient production of biological energy [32].

Eukaryotic protein Fumarate acyl‐coenzyme A hydrolase domain‐containing protein 1 (FAHD1) plays a crucial role in regulating the integration of the tricarboxylic acid cycle as a mitochondrial oxaloacetate decarboxylase (ODx) [33]. In this study, the upregulation of FAHD1 expression suggests a reduced flux of oxaloacetate into the TCA cycle, which might lead to inhibition of the TCA cycle. The resulting pyruvate might further produce ketone bodies that enter the bloodstream. Similarly, MDH1 and MDH2 are two important malate dehydrogenases. MDH1 is located in the cytosol, while MDH2 is found in the mitochondria. The main function is to oxidize malate to oxaloacetate, which is crucial for the TCA cycle [34]. Additionally, MDH1 is one of the key enzymes in biological carbohydrate metabolism and plays an important role in oxidative stress processes. It is involved in the malate–aspartate shuttle, coordinating glycolysis and mitochondrial respiration to reduce intracellular ROS levels [35]. This study found that the expression of MDH1 and MDH2 was upregulated, which might suggest an increase in oxaloacetate synthesis in ketosis cows, indicating that the body is in an oxidative stress state.

In this study, the levels of upstream metabolites related to the TCA cycle, such as L‐glutamate, proline, lysine, and threonine, increased, which might be associated with the impairment of the TCA cycle. Additionally, in the muscle fibers of cows with postpartum ketosis, the TCA cycle is inhibited, suggesting disrupted energy metabolism, increased oxidative stress, and excessive production of BHBA. However, due to the limited sample size and sampling constraints (such as the absence of pre‐calving muscle samples), it can only be speculated that ROS damage exists in the muscle tissue of cows with ketosis, and further experiments are needed to verify this.

### 4.3 Lipid Metabolism

The PPAR signaling pathway is an important metabolic regulatory pathway that primarily participates in physiological processes such as lipid metabolism, glucose metabolism, and inflammatory responses. PPARα is primarily expressed in the liver, heart, and skeletal muscle, regulating the oxidation and metabolism of fatty acids, playing a crucial role in energy metabolism [36]. In this study, we found that 10 DSPs were upregulated in the CK group of cows. These differential proteins are associated with multiple functions, including fatty acid transport, fatty acid oxidation, adipocyte differentiation, and gluconeogenesis. These results suggest that lipid metabolism in the muscle tissue of cows after calving might be upregulated via the PPAR signaling pathway [37, 38]. The APOA1, FABP_3_, FABP_4_, and FABP_5_ were upregulated in the CK group, which might indicate an increase in lipid transport, fat formation, and intramuscular lipid metabolism in cows. This finding is consistent with previous results regarding the impact of peripartum cows on lipid metabolism in the liver [39, 40, 41].

FABPs are a family of intracellular lipid chaperones that coordinate intracellular lipid responses by binding and redistributing intracellular fatty acids [42]. PPARγ regulates the expression of genes related to lipid metabolism, promoting fatty acid storage and reducing fatty acid oxidation. It affects systemic lipid levels by modulating the synthesis of FA and TG [43]. Therefore, this study found that ketosis cows might provide the necessary energy for the body post‐calving by increasing the transport and metabolic activity of fatty acids within muscle tissue and by forming substances such as triglycerides through the increase of intramuscular adipocytes to meet energy demands. However, due to the reduction in the TCA cycle, processes like ATP production are suppressed, which might indicate that simply increasing fatty acid metabolism does not fundamentally resolve the energy demand issue. Other approaches, such as improving muscle fiber condition and reducing ROS production, are also necessary to address energy deficiency. However, the specific metabolic and improvement mechanisms still require further experimental validation.

### 4.4 OXPHOS and Oxidative Status

Although the mobilization of lipids and proteins can provide substrates for energy generation to temporarily meet the energy demands during lactation, this process might simultaneously produce some ROS. ROS (superoxide [O^2−^] and hydrogen peroxide [H_2_O_2_]) are primarily generated during oxidative phosphorylation, the TCA cycle, and the oxidation of intracellular fatty acids [44, 45]. In this study, the increase in arachidonic acid and upstream metabolites, n‐arachidonoyl dopamine and arachidonoylglycine, might indirectly contribute to higher ROS levels through certain biological pathways, such as inflammatory responses. Previous studies have shown that severe redox imbalance can lead to oxidative stress in transition cows, reducing resistance to other diseases. To combat the O^2−^ generated by the body, SOD3 is upregulated in the muscle tissue of CK cows. SOD3 belongs to the superoxide dismutase (SOD) family and is the main enzyme that metabolizes ROS by converting superoxide into hydrogen peroxide [46]. At the same time, this study also found that PRDX1, LOD4, and DHRS11G were upregulated in the CK group. These proteins effectively remove H_2_O_2_, protecting cells from oxidative damage and maintaining intracellular redox balance [47].

The results of this study found that NUDFs3, NDUFV2, and NDUFA5, components of mitochondrial complex I in the electron transport chain, were upregulated in the CK. Mitochondrial complex I is a key entry point of the electron transport chain, accepting electron donors NADH generated from the TCA cycle, and then oxidizing them to initiate electron transfer [48]. However, due to the downregulation of SDHA and SDHB, the function of complex II (succinate dehydrogenase) might be inhibited. SDHA and SDHB are enzymes involved in cellular respiration and energy metabolism, serving as the two hydrophilic subunits of the succinate dehydrogenase (SDH) complex, a key component of the mitochondrial respiratory chain. As another subunit of SDH, the main role of SDHB is to participate in electron transfer, passing electrons from SDHA to coenzyme Q (CoQ), thus entering the respiratory chain [49]. Therefore, the reduction of these two enzymes might lead to decreased TCA cycle activity, impaired oxidative phosphorylation, and disruptions in bioenergetic metabolism. This impairment in succinate metabolism might reduce the efficiency of the electron transport chain, leading to increased ROS production, which triggers oxidative stress and damages cells. In complex III, electrons are transferred from one cytochrome to another through iron–sulfur proteins, ultimately being passed to Cytc. QCR6 and QCR7 are responsible for transferring electrons from CoQ to Cytc, facilitating the reduction of oxygen and energy production [50]. Therefore, in the muscle cells of ketosis cows, the function of complex III might be impaired, leading to a decreased efficiency of electron transfer from coenzyme Q to cytochrome c, which in turn affects the overall function of the electron transport chain. This might also result in increased electron leakage and the generation of ROS, triggering oxidative stress and causing damage to the cells.

## 5 Conclusions

Omics data suggest that the impaired TCA cycle function in the muscle tissue of ketosis cows might be one of the reasons for the inability to meet the energy demands of ketosis cows. Although the total pyruvate content decreased, fatty acid oxidation and the production of pyruvate from oxaloacetate might further increase the BHBA content in muscle tissue through the synthesis of ketone bodies. In addition, the mobilization of fatty acids, increased carbohydrate metabolism, and impaired function of complexes II and III in the mitochondrial respiratory chain suggest an elevated oxidative state in muscle tissue; however, further experiments are needed to confirm. These data analyze the physiological metabolic mechanisms of muscle tissue in cows with ketosis from the perspectives of proteomics and metabolomics, providing preliminary insights into the metabolic mechanisms of BHBA in muscle. This offers a theoretical basis for understanding the specific processes in muscle tissue during this period and encourages consideration of how to reduce the incidence of ketosis in cows by examining the catabolism of BHBA in muscle.

## Disclosure

Authors declare no off‐label use of antimicrobials.

## Ethics Statement

A**l**l animal experiments were performed under the direction of the Institutional Animal Care and Use Committee from the College of Animal Science and Technology, Sichuan Agricultural University, China (Certification No. SYXK2019‐187; Approval time: 2019/1/29). Authors declare human ethics approval was not needed.

## Conflicts of Interest

The authors declare no conflicts of interest.

## Supporting information


**Table S1:** Characteristics of the Chinese Holstein dairy cows used in the study.

**Table S2:** Article Specific information of proteins used in WB assay.

**Table S3:** Produced metabolite screening for pathway assessment recognized by GC/MS in the muscle biopsies of CK and Con (*n* = 6). FC = fold change; VIP = variable importance projection.

**Table S4:** Produced metabolite screening for pathway assessment recognized by GC/MS in the muscle biopsies of CK and Con (*n* = 6). FC = fold change; RT = retention time; m/z = mass to charge ratio; VIP = variable importance projection. Con and CK represent the healthy and clinical ketosis cows, respectively. + and −: abundance increased and decreased. *P*‐values were determined utilizing the student's *t*‐test between the Con and CK.

**Table S5:** Differentially produced metabolite screening for pathway assessment recognized by GC/MS in the muscle biopsies of CK and Con (*n* = 6). FC = fold change; RT = retention time; m/z = mass to charge ratio; VIP = variable importance projection. Con and CK represent the healthy and clinical ketosis cows, respectively. + and −: abundance increased and decreased.
Padj is the *P* value corrected by FDR = 0.05.

**Table S6:** Differentially produced protein screening for pathway assessment recognized by GC/MS in the muscle biopsies of CK and Con (*n* = 6).

## References

[1] A. W. Bell , “Regulation of Organic Nutrient Metabolism During Transition From Late Pregnancy to Early Lactation,” Journal of Animal Science 73, no. 9 (1995): 2804–2819. 8582872 10.2527/1995.7392804x

[2] A. A. Adewuyi , E. Gruys , and F. J. C. M. Van Eerdenburg , “Non Esterified Fatty Acids (NEFA) in Dairy Cattle. A Review,” Veterinary Quarterly 27, no. 3 (2005): 117–126. 16238111 10.1080/01652176.2005.9695192

[3] J. C. Newman and E. V. Ketone , “Ketone Bodies as Signaling Metabolites,” Trends in Endocrinology and Metabolism: TEM 25, no. 1 (2014): 42–52. 24140022 10.1016/j.tem.2013.09.002 PMC4176946

[4] T. F. Duffield , K. D. Lissemore , B. W. Mcbride , and K. E. Leslie , “Impact of Hyperketonemia in Early Lactation Dairy Cows on Health and Production,” Journal of Dairy Science 92, no. 2 (2009): 571–580. 19164667 10.3168/jds.2008-1507

[5] J. A. Mcart , D. V. Nydam , and G. R. Oetzel , “Epidemiology of Subclinical Ketosis in Early Lactation Dairy Cattle,” Journal of Dairy Science 95, no. 9 (2012): 5056–5066. 22916909 10.3168/jds.2012-5443

[6] J. M. Cainzos , C. Andreu‐Vazquez , M. Guadagnini , A. Rijpert‐Duvivier , and T. Duffield , “A Systematic Review of the Cost of Ketosis in Dairy Cattle,” Journal of Dairy Science 105, no. 7 (2022): 6175–6195. 35534272 10.3168/jds.2021-21539

[7] D. Raboisson , M. Mounié , E. Khenifar , and E. Maigné , “The Economic Impact of Subclinical Ketosis at the Farm Level: Tackling the Challenge of Over‐Estimation due to Multiple Interactions,” Preventive Veterinary Medicine 122, no. 4 (2015): 417–425. 26276398 10.1016/j.prevetmed.2015.07.010

[8] C. Loiklung , P. Sukon , and C. Thamrongyoswittayakul , “Global Prevalence of Subclinical Ketosis in Dairy Cows: A Systematic Review and Meta‐Analysis,” Research in Veterinary Science 144 (2022): 66–76. 35077992 10.1016/j.rvsc.2022.01.003

[9] C. Guo , D. Sun , X. Wang , and S. Mao , “A Combined Metabolomic and Proteomic Study Revealed the Difference in Metabolite and Protein Expression Profiles in Ruminal Tissue From Goats Fed Hay or High‐Grain Diets,” Frontiers in Physiology 10 (2019): 66. 30800073 10.3389/fphys.2019.00066 PMC6375843

[10] U. K. Sundekilde , N. A. Poulsen , L. B. Larsen , and H. C. Bertram , “Nuclear Magnetic Resonance Metabonomics Reveals Strong Association Between Milk Metabolites and Somatic Cell Count in Bovine Milk,” Journal of Dairy Science 96, no. 1 (2013): 290–299. 23182357 10.3168/jds.2012-5819

[11] Z. L. Wu , S. Y. Chen , S. Hu , X. Jia , J. Wang , and S.‐J. Lai , “Metabolomic and Proteomic Profiles Associated With Ketosis in Dairy Cows,” Frontiers in Genetics 11 (2020): 551587. 33391334 10.3389/fgene.2020.551587 PMC7772412

[12] B. He , Z. Huang , C. Huang , and E. C. Nice , “Clinical Applications of Plasma Proteomics and Peptidomics: Towards Precision Medicine,” Proteomics. Clinical Applications 16, no. 6 (2022): e2100097. 35490333 10.1002/prca.202100097

[13] C. Xu and Z. Wang , “Comparative Proteomic Analysis of Livers From Ketotic Cows,” Veterinary Research Communications 32, no. 3 (2008): 263–273. 18080212 10.1007/s11259-007-9028-4

[14] Q. Xu , X. Li , L. Ma , et al., “Adipose Tissue Proteomic Analysis in Ketotic or Healthy Holstein Cows in Early Lactation,” Journal of Animal Science 97, no. 7 (2019): 2837–2849. 31267132 10.1093/jas/skz132 PMC6606492

[15] Y. Ma , S. Feng , X. Wang , et al., “Exploration of Exosomal microRNA Expression Profiles in Pigeon ‘Milk’ During the Lactation Period,” BMC Genomics 19, no. 1 (2018): 828. 30458711 10.1186/s12864-018-5201-0 PMC6245878

[16] X. Gao , L. Jian , L. Zhang , et al., “Perilipin 5 Protects the Mitochondrial Oxidative Functions and Improves the Alcoholic Liver Injury in Mice,” Liver International 44, no. 2 (2024): 357–369. 37933091 10.1111/liv.15775

[17] W. B. Dunn , D. Broadhurst , P. Begley , et al., “Procedures for Large‐Scale Metabolic Profiling of Serum and Plasma Using Gas Chromatography and Liquid Chromatography Coupled to Mass Spectrometry,” Nature Protocols 6, no. 7 (2011): 1060–1083. 21720319 10.1038/nprot.2011.335

[18] J. Wang , T. Zhang , X. Shen , et al., “Serum Metabolomics for Early Diagnosis of Esophageal Squamous Cell Carcinoma by UHPLC‐QTOF/MS,” Metabolomics 12, no. 7 (2016): 1–10.

[19] J. Zhang , H. T. Shi , Y. C. Wang , et al., “Carbohydrate and Amino Acid Metabolism and Oxidative Status in Holstein Heifers Precision‐Fed Diets With Different Forage to Concentrate Ratios,” Animal 14, no. 11 (2020): 2315–2325. 32602427 10.1017/S1751731120001287

[20] M. Kanehisa , M. Furumichi , M. Tanabe , Y. Sato , and K. Morishima , “KEGG: New Perspectives on Genomes, Pathways, Diseases and Drugs,” Nucleic Acids Research 45, no. 1 (2017): D353–D361. 27899662 10.1093/nar/gkw1092 PMC5210567

[21] J. K. Drackley , T. R. Overton , and G. N. Douglas , “Adaptations of Glucose and Long‐Chain Fatty Acid Metabolism in Liver of Dairy Cows During the Periparturient Period,” Journal of Dairy Science 84 (2001): E100–E112.

[22] A. T. Van Knegsel , H. Brand , J. Dijkstra , et al., “Dietary Energy Source in Dairy Cows in Early Lactation: Energy Partitioning and Milk Composition,” Journal of Dairy Science 90, no. 3 (2007): 1467–1476. 17297120 10.3168/jds.S0022-0302(07)71632-6

[23] L. Doepel , H. Lapierre , and J. J. Kennelly , “Peripartum Performance and Metabolism of Dairy Cows in Response to Prepartum Energy and Protein Intake,” Journal of Dairy Science 85, no. 9 (2002): 2315–2334. 12362465 10.3168/jds.S0022-0302(02)74312-9

[24] G. Højfeldt , T. Sorenson , A. Gonzales , M. Kjaer , J. L. Andersen , and A. L. Mackey , “Fusion of Myofibre Branches is a Physiological Feature of Healthy Human Skeletal Muscle Regeneration,” Skeletal Muscle 13, no. 1 (2023): 13. 37573332 10.1186/s13395-023-00322-2 PMC10422711

[25] T. A. Järvinen , T. L. Järvinen , M. Kääriäinen , H. Kalimo , and M. Järvinen , “Muscle Injuries: Biology and Treatment,” American Journal of Sports Medicine 33, no. 5 (2005): 745–764. 15851777 10.1177/0363546505274714

[26] S. K. Powers , D. Criswell , J. Lawler , et al., “Influence of Exercise and Fiber Type on Antioxidant Enzyme Activity in Rat Skeletal Muscle,” American Journal of Physiology 266, no. 2 (1994): R375–R380. 8141392 10.1152/ajpregu.1994.266.2.R375

[27] J. O. Marx , M. C. Olsson , and L. Larsson , “Scaling of Skeletal Muscle Shortening Velocity in Mammals Representing a 100,000‐Fold Difference in Body Size,” European Journal of Physiology 452, no. 2 (2006): 222–230. 16333661 10.1007/s00424-005-0017-6

[28] F. J. Corpas , J. B. Barroso , L. M. Sandalio , J. M. Palma , J. A. Lupiánez , and L. A. del Rıo , “Peroxisomal NADP‐Dependent Isocitrate Dehydrogenase. Characterization and Activity Regulation During Natural Senescence,” Plant Physiology 121, no. 3 (1999): 921–928. 10557241 10.1104/pp.121.3.921 PMC59455

[29] J. G. Mccormack and R. M. Denton , “The Effects of Calcium Ions and Adenine Nucleotides on the Activity of Pig Heart 2‐Oxoglutarate Dehydrogenase Complex,” Biochemical Journal 180, no. 3 (1979): 533–544. 39549 10.1042/bj1800533 PMC1161091

[30] J. A. Ronchi , T. R. Figueira , F. G. Ravagnani , H. C. F. Oliveira , A. E. Vercesi , and R. F. Castilho , “A Spontaneous Mutation in the Nicotinamide Nucleotide Transhydrogenase Gene of C57BL/6J Mice Results in Mitochondrial Redox Abnormalities,” Free Radical Biology & Medicine 63 (2013): 446–456. 23747984 10.1016/j.freeradbiomed.2013.05.049

[31] B. Glancy and R. S. Balaban , “Protein Composition and Function of Red and White Skeletal Muscle Mitochondria,” American Journal of Physiology‐Cell Physiology 300, no. 6 (2011): C1280–C1290. 21289287 10.1152/ajpcell.00496.2010 PMC3118618

[32] P. Rustin and A. Rötig , “Inborn Errors of Complex II—Unusual Human Mitochondrial Diseases,” Biochimica et Biophysica Acta 1553, no. 1 (2002): 117–122. 11803021 10.1016/s0005-2728(01)00228-6

[33] H. Pircher , S. Von Grafenstein , T. Diener , et al., “Identification of FAH Domain‐Containing Protein 1 (FAHD1) as Oxaloacetate Decarboxylase,” Journal of Biological Chemistry 290, no. 11 (2015): 6755–6762. 25575590 10.1074/jbc.M114.609305 PMC4358102

[34] M. Wang , C. Zhou , L. Yu , et al., “Upregulation of MDH1 Acetylation by HDAC6 Inhibition Protects Against Oxidative Stress‐Derived Neuronal Apoptosis Following Intracerebral Hemorrhage,” Cellular and Molecular Life Sciences 79, no. 7 (2022): 356. 35678904 10.1007/s00018-022-04341-y PMC11073123

[35] E. Y. Kim , W. K. Kim , H. J. Kang , et al., “Acetylation of Malate Dehydrogenase 1 Promotes Adipogenic Differentiation via Activating Its Enzymatic Activity,” Journal of Lipid Research 53, no. 9 (2012): 1864–1876. 22693256 10.1194/jlr.M026567 PMC3413227

[36] A. Z. Mirza , I. Althagafi , and H. Shamshad , “Role of PPAR Receptor in Different Diseases and Their Ligands: Physiological Importance and Clinical Implications,” European Journal of Medicinal Chemistry 166 (2019): 502–513. 30739829 10.1016/j.ejmech.2019.01.067

[37] F. Hong , S. Pan , Y. Guo , P. Xu , and Y. Zhai , “PPARs as Nuclear Receptors for Nutrient and Energy Metabolism,” Molecules 24, no. 14 (2019): 2545. 31336903 10.3390/molecules24142545 PMC6680900

[38] H. Shi , J. Zhang , S. Li , et al., “Effects of a Wide Range of Dietary Forage‐To‐Concentrate Ratios on Nutrient Utilization and Hepatic Transcriptional Profiles in Limit‐Fed Holstein Heifers,” BMC Genomics 19, no. 1 (2018): 148. 29454312 10.1186/s12864-018-4529-9 PMC5816523

[39] J. Zhang , N. Gaowa , Y. Wang , et al., “Complementary Hepatic Metabolomics and Proteomics Reveal the Adaptive Mechanisms of Dairy Cows to the Transition Period,” Journal of Dairy Science 106, no. 3 (2023): 2071–2088. 36567250 10.3168/jds.2022-22224

[40] Y. Jiang , J. Liu , H. Liu , et al., “miR‐381‐3p Inhibits Intramuscular Fat Deposition Through Targeting FABP3 by ceRNA Regulatory Network,” Biology 11, no. 10 (2022): 1497. 36290402 10.3390/biology11101497 PMC9598794

[41] A. S. Bhale and K. Venkataraman , “Leveraging Knowledge of HDLs Major Protein ApoA1: Structure, Function, Mutations, and Potential Therapeutics,” Biomedicine & Pharmacotherapy 154 (2022): 113634. 36063649 10.1016/j.biopha.2022.113634

[42] C. M. Scifres , B. Chen , D. M. Nelson , and Y. Sadovsky , “Fatty Acid Binding Protein 4 Regulates Intracellular Lipid Accumulation in Human Trophoblasts,” Journal of Clinical Endocrinology and Metabolism 96, no. 7 (2011): E1083–E1091. 21525163 10.1210/jc.2010-2084 PMC3135200

[43] N. L. Bertschi , O. Steck , F. Luther , et al., “PPAR‐γ Regulates the Effector Function of Human T Helper 9 Cells by Promoting Glycolysis,” Nature Communications 14, no. 1 (2023): 2471. 10.1038/s41467-023-38233-x PMC10148883 37120582

[44] P. F. Surai , I. Kochish , V. I. Fisinin , I. I. Kochish , and D. T. Juniper , “Revisiting Oxidative Stress and the Use of Organic Selenium in Dairy Cow Nutrition,” Animals 9, no. 7 (2019): 462. 31331084 10.3390/ani9070462 PMC6680431

[45] G. A. Contreras and L. M. Sordillo , “Lipid Mobilization and Inflammatory Responses During the Transition Period of Dairy Cows,” Comparative Immunology, Microbiology and Infectious Diseases 34, no. 3 (2011): 281–289. 21316109 10.1016/j.cimid.2011.01.004

[46] I. N. Zelko , T. J. Mariani , and R. J. Folz , “Superoxide Dismutase Multigene Family: A Comparison of the CuZn‐SOD (SOD1), Mn‐SOD (SOD2), and EC‐SOD (SOD3) Gene Structures, Evolution, and Expression,” Free Radical Biology and Medicine 33, no. 3 (2002): 337–349. 12126755 10.1016/s0891-5849(02)00905-x

[47] A. Moretton , S. Kourtis , A. Gañez Zapater , et al., “A Metabolic Map of the DNA Damage Response Identifies PRDX1 in the Control of Nuclear ROS Scavenging and Aspartate Availability,” Molecular Systems Biology 19, no. 7 (2023): e11267. 37259925 10.15252/msb.202211267 PMC10333845

[48] H. Pelicano , W. Lu , Y. Zhou , et al., “Mitochondrial Dysfunction and Reactive Oxygen Species Imbalance Promote Breast Cancer Cell Motility Through a CXCL14‐Mediated Mechanism,” Cancer Research 69, no. 6 (2009): 2375–2383. 19276362 10.1158/0008-5472.CAN-08-3359 PMC2760349

[49] R. D. Guzy , B. Sharma , E. Bell , N. S. Chandel , and P. T. Schumacker , “Loss of the SdhB, but Not the SdhA, Subunit of Complex II Triggers Reactive Oxygen Species‐Dependent Hypoxia‐Inducible Factor Activation and Tumorigenesis,” Molecular and Cellular Biology 28, no. 2 (2008): 718–731. 17967865 10.1128/MCB.01338-07 PMC2223429

[50] L. Zeng , Y. Huang , J. Tan , et al., “QCR7 Affects the Virulence of Candida Albicans and the Uptake of Multiple Carbon Sources Present in Different Host Niches,” Frontiers in Cellular and Infection Microbiology 13 (2023): 1136698. 36923588 10.3389/fcimb.2023.1136698 PMC10009220

